# HEK293 cell response to static magnetic fields via the radical pair mechanism may explain therapeutic effects of pulsed electromagnetic fields

**DOI:** 10.1371/journal.pone.0243038

**Published:** 2020-12-03

**Authors:** Marootpong Pooam, Nathalie Jourdan, Mohamed El Esawi, Rachel M. Sherrard, Margaret Ahmad

**Affiliations:** 1 Sorbonne Université – CNRS, UMR8256 - IBPS, Paris, France; 2 Department of Biology, Faculty of Science, Naresuan University, Phitsanulok, Thailand; 3 Botany Department, Faculty of Science, Tanta University, Tanta, Egypt; 4 Xavier University, Cincinnati, Ohio, United States of America; Xiangtan University, CHINA

## Abstract

PEMF (Pulsed Electromagnetic Field) stimulation has been used for therapeutic purposes for over 50 years including in the treatment of memory loss, depression, alleviation of pain, bone and wound healing, and treatment of certain cancers. However, the underlying cellular mechanisms mediating these effects have remained poorly understood. In particular, because magnetic field pulses will induce electric currents in the stimulated tissue, it is unclear whether the observed effects are due to the magnetic or electric component of the stimulation. Recently, it has been shown that PEMFs stimulate the formation of ROS (reactive oxygen species) in human cell cultures by a mechanism that requires cryptochrome, a putative magnetosensor. Here we show by qPCR analysis of ROS-regulated gene expression that simply removing cell cultures from the Earth’s geomagnetic field by placing them in a Low-Level Field condition induces similar effects on ROS signaling as does exposure of cells to PEMF. This effect can be explained by the so-called Radical Pair mechanism, which provides a quantum physical means by which the rates and product yields (e.g. ROS) of biochemical redox reactions may be modulated by magnetic fields. Since transient cancelling of the Earth’s magnetic field can in principle be achieved by PEMF exposure, we propose that the therapeutic effects of PEMFs may be explained by the ensuing modulation of ROS synthesis. Our results could lead to significant improvements in the design and therapeutic applications of PEMF devices.

## Introduction

All life on Earth is exposed to a magnetic field generated through the motion of molten metal at the Earth’s core. This so-called geomagnetic field is a directional force that moves outward from near the South Pole, then curves upwards, and re-enters the Earth near the North Pole [1]. The greatest benefit to living organisms of the geomagnetic field is to deflect high-energy cosmic rays emitted by solar wind, which would otherwise make life on earth impossible. However, the geomagnetic field in and of itself also has effects on living systems. For example, studies in numerous organisms indicate that the magnetic field can be used as a source of directional information, as well as inducing cellular and physiological consequences [1–3]. Because these biological effects are generally weak, often of unknown mechanism, and all requiring carefully controlled conditions to reproducibly demonstrate, there has been considerable lack of clarity in the field.

However, in spite of the many unknowns, the application of magnetic field pulses has been empirically and reliably proven an effective therapeutic tool in the treatment of human disease. In particular, Pulsed Electromagnetic Field (PEMF) therapy has been used. PEMF therapy involves the non-invasive exposure of parts of the human body to low intensity pulsed electromagnetic fields in the 10–300 Hz range, typically for a few minutes a day over a period of several days to several weeks [3]. Clinical studies have shown the effectiveness of PEMF in treating such conditions as arthritis [4, 5], chronic pain [6, 7], bone injury [8–10], wound healing [11–15], and lupus erythematosus [16]. All of these conditions furthermore involve resolution of chronic or acute inflammation, which is sensitive to intracellular concentrations of ROS (reactive oxygen species). Although many changes in cellular and biochemical markers induced by PEMF exposure have been documented [e.g. in 17, 18], the molecular mechanisms underlying therapeutic effects of PEMF stimulation remains unknown.

PEMF signals comprise both magnetic and electrical field components, with considerable variability imposed by signal shapes, intensities, and frequencies [19, 20]. Although many studies have suggested that PEMF alters cellular function through the action of induced electrical currents, others have suggested that the magnetic field component of PEMF stimulation is what induces the physiological effects [21, 22]. This latter possibility has received support from a recent study on the response characteristics of human cell cultures to 10Hz PEMF exposure, in which a putative magnetic field receptor, known as cryptochrome, has been implicated [23]. A rapid increase in cellular concentration of ROS, with accompanying changes in gene expression, were observed in response to the PEMF signal [23]. These experiments do not exclude that the induced electric fields may trigger the biological response. However, they nonetheless raise the intriguing possibility that therapeutic PEMF effects could be mediated through the same underlying mechanisms as are responsible for biological responses to static magnetic fields, which do not generate electric currents.

Currently, there are two well-characterized biological magnetosensing mechanisms. One of these involves ferromagnetite, an iron-based compound, which can be attached to cellular structures or proteins to enable organisms to orient in the direction of the magnetic north pole [24, 25]. In addition, there has been accumulating evidence of biological magnetosensing through the so-called ‘Radical Pair Mechanism’ [26]. This mechanism involves quantum physical effects of electromagnetic fields on electron spin of reaction intermediates formed during susceptible enzymatic reactions. Flavoenzyme-based redox reactions have been proposed as particularly likely candidates for these quantum forces. In summary, the effect of a weak magnetic field would be to alter rates of enzyme-catalyzed chemical reactions within the cell. These could be of metabolic enzymes, resulting in altered metabolism or altered rates of biosynthesis of ROS (a byproduct of redox chemistry). Intriguingly, an evolutionarily conserved flavoprotein receptor known as cryptochrome [27], also undergoes redox chemistry thought compatible with the Radical Pair Mechanism for magnetic sensitivity [28]. Cryptochrome has been linked to static field magnetosensing in many biological systems [28–30], and has also been implicated in sensitivity to PEMF exposure in human HEK cells [23] and mouse organotypic brain culture [31].

These observations thereby directly pose the question of whether the effects of PEMF stimulation result from one of the known biological magnetosensing mechanisms, instead of being an effect of induced electrical currents. A clear prediction would then be that a simple static magnetic field should provide some of the same effects as the PEMF signal on stimulation of cellular ROS.

In the present work we address this question by determining the effect on HEK293 cells in culture of exposure to a number of static magnetic field conditions. Firstly we assayed cells at 2 mT, which was the peak intensity of magnetic field output by the PEMF device used in our original study [23] showing induction of ROS. We also chose 500 μT since it induces physiological effects and modulation of cryptochrome function in a number of experimental systems including *Drosophila* behavioral assays and in plants [28, 30]. We chose a low-level field (LLF) to provide a condition where the Earth’s magnetic field is essentially removed (less than 200 nT intensity).–this condition had furthermore been previously shown to induce biosynthesis of ROS in a variety of mammalian cell cultures [32], similarly to our PEMF device [23] Finally, we chose the ambient Earth’s geomagnetic field as the control condition, which was 40 μT inside the incubator. For our biological assay, we have monitored expression of ROS-responsive genes that were up-regulated as a result of PEMF stimulation to use as markers for cellular ROS induction [23].

In summary, this study explores whether the effects of PEMF stimulation on cell cultures can be mimicked by using only static magnetic fields. A positive outcome would provide for the first time a molecular and biophysical explanation for the therapeutic effects of PEMF by known biochemical and quantum physical mechanisms.

## Materials and methods

### Cell culture and growth conditions

Methods in this study are essentially identical to those previously described [23]. HEK 293 (ATCC-CRL-1573) cells were acquired from the ATCC cell bank Briefly, human embryonic kidney (HEK) 293 cells were cultured in a CO_2_ incubator (MCO-18AC, Panasonic Biomedical, Leicestershire, UK), at 37°C and 5% CO_2_. Cells were grown in 75 ml culture flasks containing 10 ml Modified Eagle medium (MEM; Sigma, Sigma, St Louis, MO) and sub-cultured every 4 days.

### Static magnetic field and low-level field exposure conditions

The static magnetic field exposure conditions are essentially as previously described [28, 29], with the exception that all experiments were conducted in the dark, and in the CO_2_ incubator. The Control condition was the culture of HEK293 exposed to the local magnetic fields inside the CO_2_ incubator (40 μT).

The higher intensity static magnetic fields (500 μT and 2 mT) were generated by a square Helmholtz coil (20 cm per side) placed inside the incubator as described previously [23, 28, 29]. Each coil consisted of 20 turns of copper wire with the two coils separated by 11.5 cm. Current from a DC power source was used to generate parallel current to achieve the desired (500 μT or 2 mT) static MF treatment condition. The sham condition was generated by running the current through the two wire coils in antiparallel directions to cancel the magnetic fields without affecting other environmental components (e.g. temperature or vibration)–described in [23, 28].

To generate the low-level static field exposure (LLF), the samples were placed within two concentric μ-metal cylinders with walls 0.2 mm thick, as described previously [29]. The inner cylinder was 11.5 cm diameter, and the outer one was 16 cm diameter, and both were 30 cm in height. The culture dish was placed at the center of the μ-metal cylinder where the intensity of local magnetic fields was lower than 200 nT (LLF). To generate the sham condition for LLF exposure, a small round Helmholtz coil was placed inside the inner μ-metal cylinder. Each coil consisted of 20 windings of 1 mm-diameter copper wire around a plastic circular frame (10 cm diameter, at a separation of 10 cm between coils). The current provided to the coils generated a 40 μT static MF, which was local magnetic field inside the incubator. This set-up provided a control condition directly within the mu-metal cylinder.

For all Magnetic Field exposure experiments, HEK293 cells were seeded into 3.5 cm^2^ round culture-dishes and grown for 48 h. Subsequently, culture dishes were exposed to LLF, Control (40 μT), 500 μT or 2000 μT static fields for either 10 minutes or 3h, which we have previously shown increase ROS and modify gene expression [23]. In all cases cells were harvested 3 hours after initial onset of exposure. Control and sham conditions using the same cell cultures were always performed in parallel for comparative purposes.

### Quantitative RT-PCR analysis of altered gene expression

The qPCR analysis was performed as described [23]. After exposure to each treatment condition, total RNA was extracted from HEK293 cells by Total RNA Miniprep Kit (New England Biolabs). cDNA was prepared from 1 μg total RNA using SuperScript first-strand synthesis system (Thermo Fisher Scientific). Quantitative RT-PCR was performed using Luna qPCR master mix (New England Biolabs). We selected three ROS-regulated genes, KIAA1211, RPS16P5 and TAS2R19 that had been previously reported to be up-regulated after exposure to 10 Hz PEMF at 2 mT [21]. The GADPH gene was used as the reference gene. Quantitative RT-PCR was performed by Mastercycler^®^ RealPlex2 (Eppendorf). Three biological replicates were performed for each gene (N = 3). Data analysis to represent the relative expression level of genes of interest was performed as previously described [23]. Primers used for gene expression analysis are described in Table 1.

**Table 1:** List of primers used in the current study.

Gene		Primer sequence
*KIAA1211*	Forward	AGCTGGCTGTTAAGCCAAAA
	Reverse	CCTCCAGTTCTCGCCAGTAG
*RPS16P5*	Forward	TGCTAATGGCTGTGTGAAGC
	Reverse	GCCACAACAGGAAAAGGTGT
*TAS2R19*	Forward	GCAAACTGTGACCTCCTTCC
	Reverse	CGTGTCATCTGCCACAAAAC
*GADPH*	Forward	TGCACCACCAACTGCTTAGC
	Reverse	GGCATGGACTGTGGTCATGAG

### Statistical analysis

All data were analyzed by using GraphPad Prism version 7.4.2 for Mac (GraphPad Software, La Jolla California, USA). Data were analyzed for normality with the Shapiro-Wilk test. Results will be expressed as mean ± standard error of the mean (SEM). The difference between treated and control conditions for each gene were compared using One-way ANOVA followed by Tukey’s multiple comparisons test. Comparisons were of the Exposed (to a given static field condition) or Sham-exposed (to a cancelled magnetic field condition) sample relative to the Control (unexposed) sample grown at the same time from the same cell stock. Differences were considered statistically significant with a *p*-value < 0.05 (*), < 0.01 (**), <0.01 (***).

## Results

The present study seeks to establish whether exposure to a static magnetic field could promote comparable effects on cultured cells as exposure to PEMF (pulsed electromagnetic fields) and therefore clarify whether cellular effects were primarily due to the magnetic or induced-electric fields. Our assay was based on a prior study which identified marker genes that are regulated by ROS and are induced immediately following PEMF stimulation by a mechanism that implicates a known static magnetic field sensor [See supplement in ref. 23]. We monitored expression of a representative number of these genes (KIAA1211, RPS16P5, TAS2R19) as a simple and rapid assay for a biological response to static magnetic field stimulation in HEK cell cultures.

We tested a range of static magnetic field conditions: the Control condition was the local geomagnetic field measured within the incubator (40 μT); 500 μT; 2 mT and LLF (Low Level Field). LLF was at an intensity of less than 200nT, a static field which has been shown previously to induce physiological effects on biosynthesis of ROS in mammalian cell cultures [22] and so is a likely candidate for investigating PEMF induced effects.

**Fig 1:**
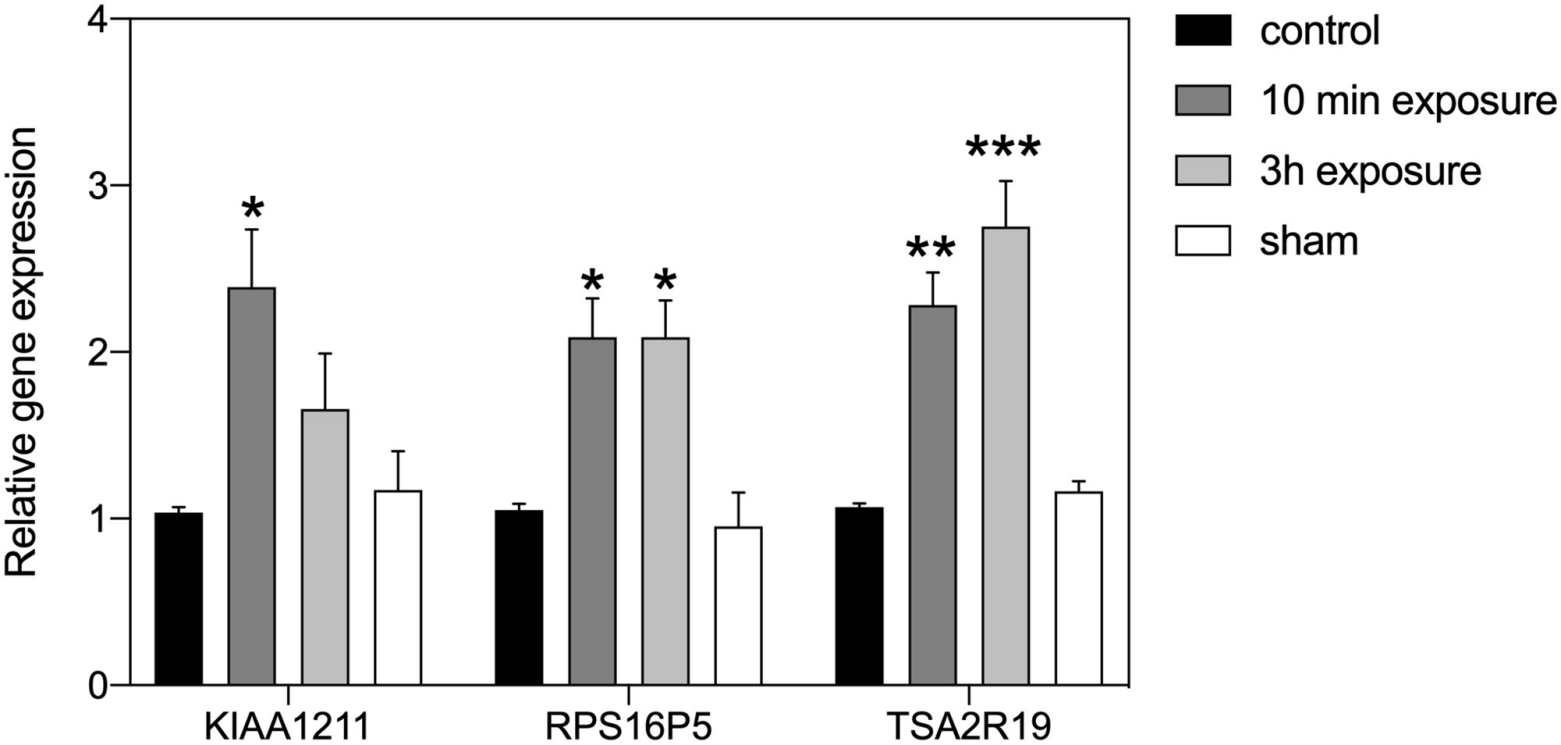
Effect of low level field (LLF) on HEK293 gene expression. The expression level of genes KIAA1211, RPS16P5 and TAS2R19 after exposure to LLF condition for 10 min (dark-grey bar) or 3h (light-grey bay) in comparison to the Control (black bar) or sham-exposed (white bar) condition. The ‘Control’ condition represents growth in the incubator at the local geomagnetic field without added applied static field. The ‘Sham’ condition was a 40 μT field produced by Helmholtz coils placed inside the Mu-metal cylinder used to generate the LLF to mimick the geomagnetic field (see methods). Data are shown as mean ± SE of three independent experiments (N = 3). The asterisks indicate significance level of the differences: *p-value < 0.1; ** *p*-value < 0.01; *** *p*-value < 0.01.

We observed different effects on gene expression according to the intensity of the static magnetic field, but not with the different durations of exposure. Significant change in gene expression was obtained from HEK cells in response to exposure to the LLF condition. Even a 10 min exposure to LLF was effective in stimulating increased expression in our selected genes. The level of induction was comparable to that obtained from these cultured cells subsequent to exposure to the 10 Hz PEMF signal [23]. Intriguingly, two of the three genes responded similarly after a 3-hour exposure period (RPS16P5, TAS2R19). However, one of the genes (KIAA1211) proved insensitive to 3 hours of continuous exposure to LLF. This suggests some degree of adaptation over the longer term, a common feature of cellular response to altered concentration of ROS [33]. The ‘Control’ and ‘Sham Exposure’ experimental conditions showed no statistical variation, indicating that the effect was indeed due to the applied static magnetic field (Fig 1).

**Fig 2:**
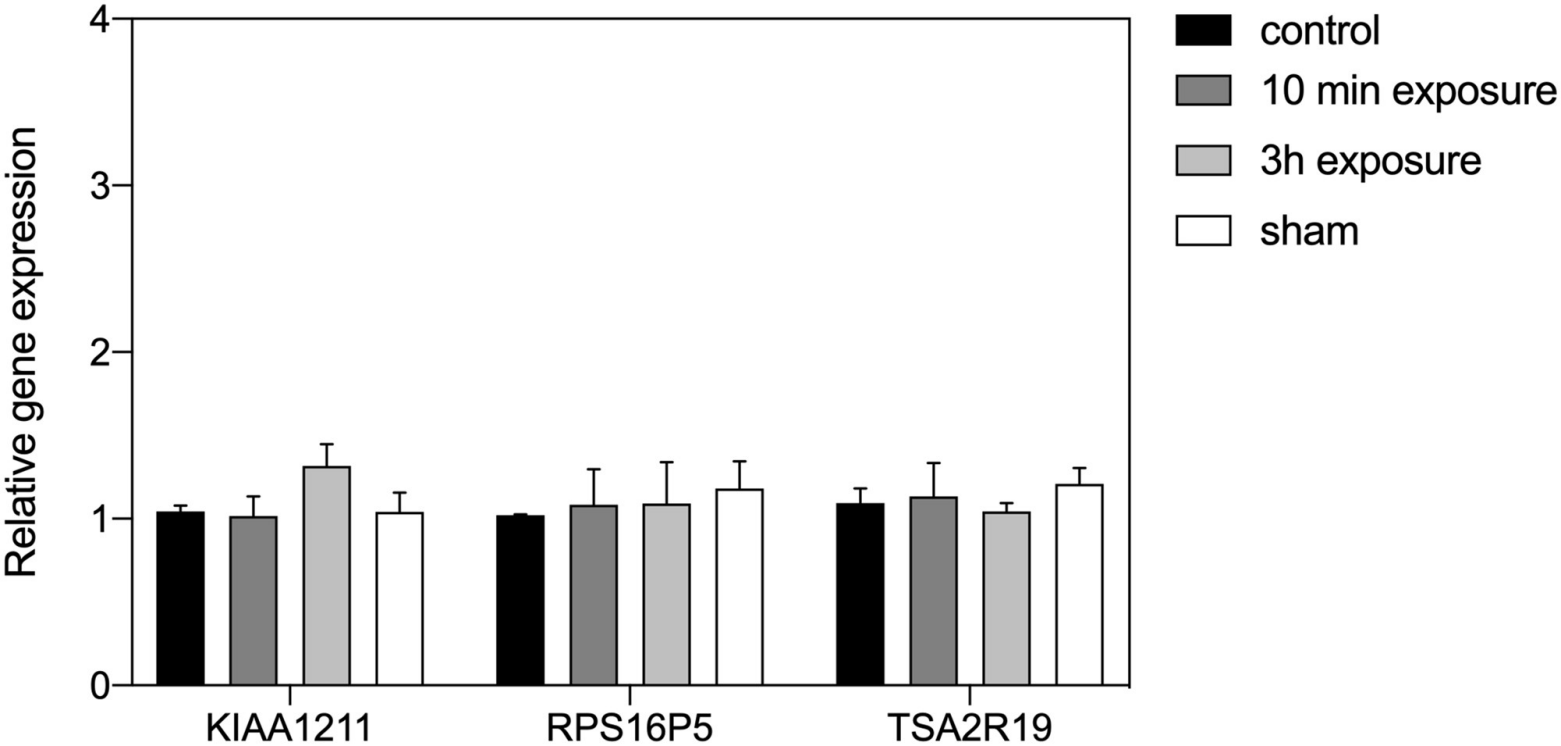
Effect of a 500 μT static magnetic field on HEK293 gene expression. The results are presented as the expression level of genes KIAA1211, RPS16P5 and TAS2R19 after exposure to 500 μT condition for 10 min (dark-grey bar) or 3h (light-grey bay) in comparison to the Control (black bar) or sham-exposed (white bar) condition. The ‘Control’ condition is the local geomagnetic field in the incubator. The ‘Sham’ condition uses a Helmholtz coil with current running in antiparallel directions to cancel the induced magnetic field (see Methods). Data are shown as mean ± SE of three independent experiments (N = 3). The asterisks indicate significance level of the differences: *p-value < 0.1; ** *p*-value < 0.01; *** *p*-value < 0.01.

Also, some studies on biological magnetosensing have used exposure conditions of 500 μT static field (roughly 10 X that of the earth’s geomagnetic field). We accordingly performed HEK cell gene expression analysis after exposure to 500 μT magnetic field intensity (Fig 2). In this case, there was no significant change in expression of any of the three tested genes as compared to Sham (mock-exposed) and Control (40 μT local field) conditions (Fig 2).

**Fig 3:**
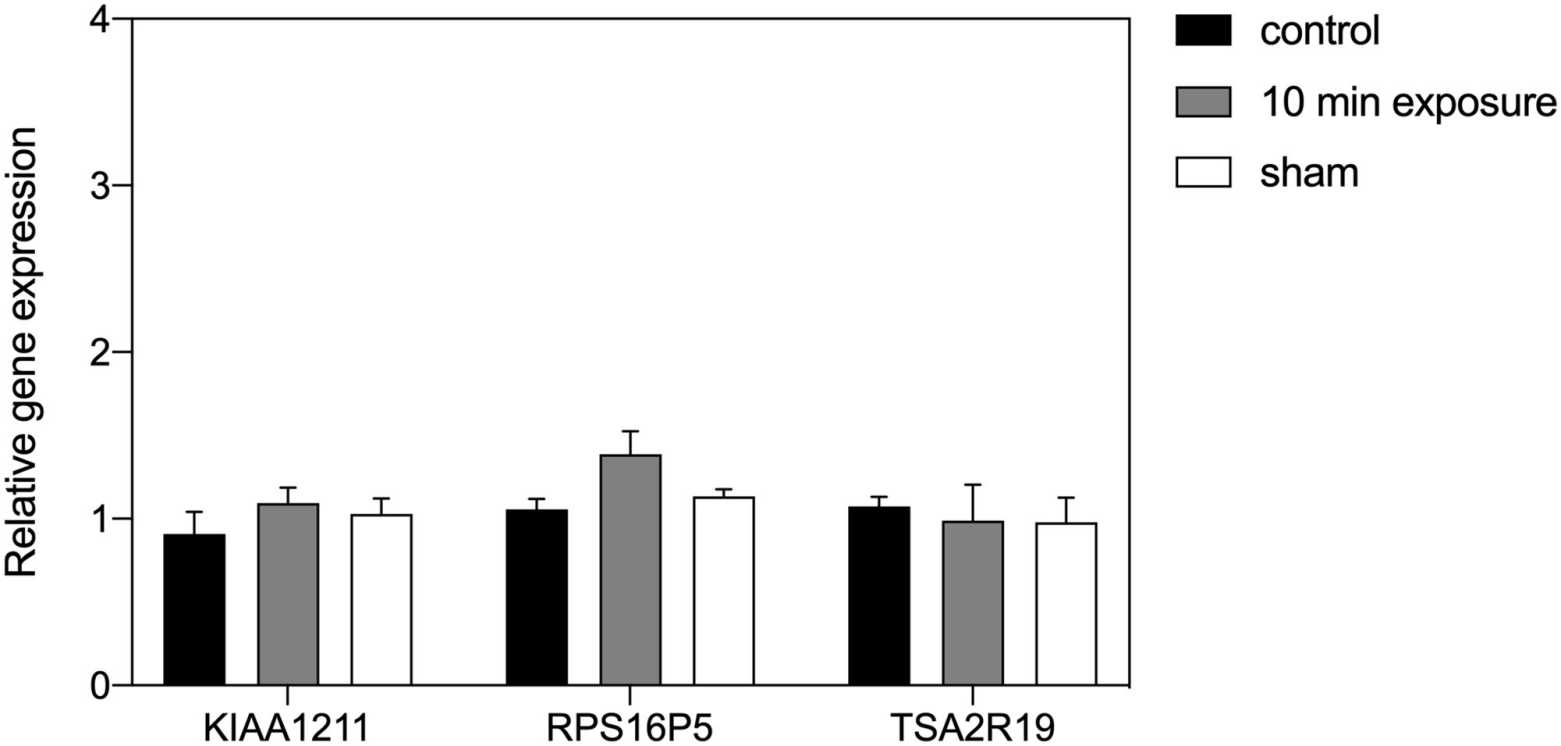
Effect of a 2 mT static magnetic field on HEK293 gene expression. The results are presented as the Relative expression of the ROS-related genes KIAA1211, RPS16P5 and TAS2R19 after 2 mT (grey bar), Control (black bar) or Sham (white bar) exposure conditions. Control cell cultures were exposed to the local magnetic field. The Sham condition was provided by the Helmholtz coil that generated the 2mT magnetic field but with antiparallel currents. Data are shown as mean ±SE of three independent experiments (N = 3).

Most experiments on static magnetic field effects on biological organisms have been observed at magnetic field strengths relatively close to that of the local geomagnetic field. In contrast, PEMF is often applied at higher intensities in the milliTesla range. However, these higher magnetic field strengths in their static form are predicted to have limited effects on biological responses by any of the known magnetosensing mechanisms [24–26]. We therefore exposed HEK293 cell cultures to a 2 mT static magnetic field (Fig 3). There was no change in gene expression to either a 10 min or 3-hour exposure at this intensity.

These results can be summarized as follows: when cells were exposed to the LLF condition, they were effectively deprived of the Earth’s magnetic field. This led to a marked increase in expression of ROS-related genes (Fig 1), indicating that physiological ROS synthesis is much lower in the presence of the local (Earth’s) geomagnetic field. Higher magnetic field strengths showed no measurable further effect on ROS-related gene expression. This indicates that regulation of cellular ROS is optimal in the presence of the geomagnetic field.

## Discussion

The goal of this study was to determine whether an underlying biological response to static magnetic fields could account for the effectiveness of PEMF as a therapeutic tool [4–13]. This was suggested by the fact that cryptochrome, a putative static magnetic field receptor, had been implicated in PEMF responses [23, 31], and by the fact that the static magnetic fields emitted transiently by PEMF devices occur within a range of intensities known to have physiological consequences [28–30]. Our initial expectation was that exposure to the higher magnetic fields (0.5 and 2 mT) might result in an enhanced biological response as compared to exposure to the earth’s geomagnetic field (40 μT). What we in fact found was that ROS–regulated gene expression was identical at all three tested static magnetic field conditions—40 μT, 0.5 mT and 2 mT (Figs 2 and 3), whereas only the absence of the magnetic field stimulated a significant increase in ROS-regulated gene expression (Fig 1). These results indicate that levels of ROS in cellular cultures are indeed regulated by the presence of a static magnetic field. Furthermore, our data suggest that ROS synthesis has an optimal biological minimum at the intensity of the Earth’s geomagnetic field, otherwise higher magnetic field strengths would have biological consequences.

To understand how a PEMF signal could create such an LLF effect, it must be considered that the magnetic field component of PEMF signals are generated by electrical currents pulsed through a coil at a defined frequency [19, 20]. During such treatments, the magnetic field is continuously increasing and decreasing in non-homogeneous directions, thereby covering a range of directional vectors and intensities (rising and falling) at rates determined by the PEMF pulse frequency and signal shape. It must also be considered that magnetic fields are additive and therefore can cancel each other out when coming from different directions at comparable intensities. To generate a Low-Level Field effect, the magnetic field emitted by the PEMF coil must at certain instants be in opposing orientation and of suitable intensity to cancel the static geomagnetic field in the incubator (around 40 μT). Over a prolonged PEMF exposure period, all of the susceptible receptor molecules throughout the sample would be intermittently exposed to the LLF.

Given the assumption that PEMF exposure can achieve a transient decline in the Earth’s magnetic field, then the PEMF effect on cellular ROS stimulation can now be explained by the ‘Radical Pair Mechanism’ of biological magnetosensing. In this mechanism, magnetic fields act on radical pairs formed by biological receptors as extremely short-lived reaction intermediates [26]. The magnetic field will then either accelerate or decrease the ultimate reaction rate and reaction product formation. Indeed, the Radical Pair mechanism has been implicated in a number of studies probing the modulation of H_2_0_2_ subsequent to LLF exposure in cellular cultures [34–36]. Transient static LLFs formed in the course of PEMF stimulation could thereby significantly impact on rates of ROS formation in cell culture. For instance, if each flavoprotein receptor molecule received a single transient LLF stimulus at least once during the (10 min or 3 hr) PEMF exposure period, the triggered change in biological activity would cause a measurable physiological change in ROS.

In this context, an important feature of the Radical Pair mechanism is that quantum physical effects of the magnetic field on electron spin chemistry are predicted to operate in a narrow range and reach a plateau beyond which further increase in magnetic field intensity could have no further effect on product yields [26]. In agreement with such a mechanism, our experiments shown no difference in biological response at Earth magnetic field (40 μT) as compared to higher magnetic field strengths (0.5 and 2 mT)–see Figs 2 and 3. Only a decrease in the magnetic field intensity (ie the LLF condition in our experiments) was able to induce measurable effects on the biological response, Our results are therefore consistent with the cellular reaction rates generating ROS being tuned to reach an optimal minimum (the physiologically most desirable steady state) near the intensity of the Earth’s magnetic field.

From a mechanistic perspective, our findings are consistent with a mode of action whereby modest, transient, changes in cellular ROS such as induced by PEMF devices [23] provide an explanation for their beneficial effects and healing functions [23]. These concentrations of ROS are much lower than those that induce damage known as oxidative stress [33], and instead involve a phenomenon known as ‘redox biology’ wherein cellular ROS plays a beneficial role by stimulating anti-oxidant defense and repair pathways [37]. In support of this mechanism, many pathologies on which therapeutic effects of PEMF have been documented [4–15] involve cellular mechanisms (eg. underlying anti-inflammatory effects) responsive to ROS signaling pathways [eg. 17, 18, 37–39]. This is also true for resolution of chronic and acute pain, for which PEMF therapy has been proven effective, and which has the great advantage that there are no potential negative side effects as caused by pharmaceutical remedies [40]. Since the Radical Pair Mechanism predicts only modest changes in concentrations of ROS on theoretical grounds, a means of inducing transient small increases in cellular ROS at will by appropriately altering the geomagnetic field provides a powerful paradigm for the effectiveness of PEMF devices in therapy.

Our findings indicate that effects of PEMF stimulation on gene expression can be induced through static magnetic field exposure. However, they do not exclude the possibility that the induced electrical fields generated by PEMF may also play a part. Our findings also do not exclude that cellular ROS stimulation which results either as a consequence of LLF or of PEMF exposure could occur by different underlying mechanisms. Ultimately, further research using defined and simplified PEMF signals, analysed by studying a variety of cellular signaling pathways, will be required to fully resolve these questions.

## Conclusion

In this study, we show that exposing human cell cultures to a reduced (Low Level) magnetic field replicates the effect of PEMF exposure on ROS-regulated gene expression. These observations are consistent with the Radical Pair Mechanism, which provides a quantum biological explanation for how the redox chemistry of susceptible flavoproteins can be manipulated by magnetic fields including that of the Earth. Because PEMF output produces magnetic fields with changing amplitudes and directions, we propose that PEMF exposure may transiently change the static magnetic field exposure conditions to alter ROS synthesis in cell cultures.

## Supporting information

S1 Appendix Files of raw data, means and S.E. used to build graphs in Figs 1–3. (XLSX)
